# Antihistamines improve cardiovascular manifestations and other symptoms of long-COVID attributed to mast cell activation

**DOI:** 10.3389/fcvm.2023.1202696

**Published:** 2023-07-17

**Authors:** Fabrizio Salvucci, Roberto Codella, Adriana Coppola, Irene Zacchei, Gabriella Grassi, Maria Luisa Anti, Nicolita Nitisoara, Livio Luzi, Carmine Gazzaruso

**Affiliations:** ^1^ Internal Medicine, Clinica Santa Rita del Gruppo Policlinico di Monza, Vercelli, Italy; ^2^ Department of Biomedical Sciences for Health, University of Milan, Milan, Italy; ^3^ Centre for Applied Clinical Research (Ce.R.C.A.), Istituto Clinico Beato Matteo, Vigevano, Italy; ^4^ Cardiovascular and Metabolic Department, Ticinello Cardiovascular and Metabolic Centre, Pavia, Italy; ^5^ Department of Endocrinology, IRCCS Multimedica, Milan, Italy

**Keywords:** long-COVID, histamine receptors, mast cell activation, antihistamines, treatment

## Abstract

**Introduction:**

Long-COVID is a broadly defined condition and there are no effective therapies. Cardiovascular manifestations of long-COVID include high heart rate, postural tachycardia, and palpitations. Previous studies have suggested that mast cell activation (MCA) may play a role in the pathophysiology of long-COVID, including in the mechanisms of its cardiovascular manifestations. The present study aimed to evaluate the effectiveness of a treatment with blockers of histamine receptors in patients with long-COVID who did not respond to other therapies.

**Methods:**

In all, 14 patients (F/M = 9/5; 49.5 ± 11.5 years) and 13 controls (F/M = 8/5; 47.3 ± 8.0 years) with long-COVID symptoms attributed to MCA were evaluated. Patients were treated with fexofenadine (180 mg/day) and famotidine (40 mg/day). Fatigue, brain fog, abdominal disorders, and increased heart rate were evaluated in treated and untreated patients at baseline and 20 days later.

**Results:**

Long-COVID symptoms disappeared completely in 29% of treated patients. There was a significant improvement in each of the considered symptoms (improved or disappeared) in all treated patients, and the improvement grade was significantly greater in treated patients compared to controls. No significant differences in the outcomes were observed in the controls.

**Conclusions:**

Our data confirm that histamine receptors blockade may be an effective target to successfully treat long-COVID. Our finding supports the underlying role of MCA in the pathophysiology of long-COVID.

## Introduction

Coronavirus disease 2019 (COVID-19) is a clinical condition due to a novel coronavirus called SARS-CoV-2 (severe acute respiratory syndrome coronavirus-2) (1). The COVID-19 pandemic has important clinical, economic, and social implications (2). SARS-Cov2 continues to spread globally, and it is responsible for the COVID-19 pandemic (1). Most patients with SARS-Cov2 infection are asymptomatic or have mild symptoms, but approximately 10% of patients with a SARS-Cov2 infection can experience a severe course with pneumonia, respiratory failure, thrombotic events, cardiovascular injury, and death (1).

Long-COVID is a multisystem disease that occurs in a proportion of patients with symptomatic or even asymptomatic SARS-Cov2 infection (3). Patients with long-COVID can exhibit involvement and impairment of multiple organs (3, 4). Cardiovascular, psychological, neurological, respiratory, gastrointestinal, dermatological, musculoskeletal, and generalized signs and symptoms have been described (3–9). Among cardiovascular manifestations, tachycardia, palpitations, and postural hypotension are the most common disorders (3–9). Other common symptoms are fatigue, cognitive impairment, shortness of breath, and cough (3–9). The pathophysiology of long-COVID is still unclear. Several mechanisms have been proposed: immune dysregulation, auto-immunity, endothelial dysfunction, occult viral persistence, and coagulation activation (6). It is plausible that a syndromic thread in each disorder has its specific derangement (8).

Some authors hypothesized that hyperinflammation linked to SARS-Cov2 infection might be at least partially mediated by mast cell activation (MCA) (10). In addition, it has been suggested that MCA might explain not only hyperinflammation in COVID-19 but also many symptoms of long-COVID (11). To test this hypothesis, Weinstock et al. compared patients with long-COVID, patients with mast cell activation syndrome (MCAS), and controls (12). They found that symptoms of MCAS were increased in long-COVID and mimicked the symptoms and severity observed in patients with MCAS (12). The study highlighted the main role that MCA can have in the pathophysiology of long-COVID, suggesting a route to effective therapies (12). One study evaluated retrospectively the clinical response to antihistamines in 26 patients with long-COVID treated with a combination of H1 and H2 receptor antagonists (13). Patients treated with antihistamines showed a significantly better response than the 23 untreated patients (13) The present study aimed to evaluate the effectiveness of treatment with blockers of histamine receptors on specific symptoms of long-COVID attributed to MCA, particularly on cardiovascular manifestations, in a well-characterized group of patients with long-COVID who were not responding to other therapies.

## Methods

In this retrospective study, we included a well-characterized cohort of 14 patients with long-COVID treated with histamine H1 and histamine H2 receptor blockers, because they were non-responders to other treatments and their symptoms seemed to be attributable to MCA (10–12). These 14 patients were selected according to rigorous inclusion and exclusion criteria from a large group of 373 people with long-COVID who were treated with H1 and H2 receptor blockers. To limit any interference on clinical response, the following inclusion criteria were used: (1) onset of symptomatic SARS-Cov2 infection, confirmed by RT-PCR, at least six months earlier; (2) diagnosis of long-COVID after acute COVID-19; (3) treatment for long-COVID with any therapy except antihistamines, sodium cromoglycate, and montelukast; (4) no registered benefits from previous treatment for long-COVID; and (5) presence of fatigue and at least one of the following symptoms: (a) increased heart rate (HR), (b) brain fog, and (c) abdominal disorders. The exclusion criteria were: (1) personal history of allergy and/or any disease characterized by MCA; (2) presence of any symptom among those considered in the diagnosis of long-COVID before the occurrence of COVID-19; (3) vaccination against SARS-Cov2; (4) presence of any disease other than long-COVID and any pharmacological or nutraceutical treatment; and (5) presence of symptoms not attributed to MCA by previous studies.

Fatigue was defined as severe, persistent, and disabling fatigue that can be associated with headaches, myalgia, shortness of breath, and orthostatic intolerance. Increased heart rate was defined as an HR >100 beats/min at rest or a postural tachycardia (increased HR of >30 beats per min within 5–10 min of standing). Increased heart rate can be associated with palpitations, exercise intolerance, and chest pain. Brain fog was defined as lack of concentration, dizziness and/or lightheadedness and/or confusion, memory loss, difficulty multitasking, and inability to find the right words. Gastrointestinal manifestations included nausea and/or vomiting, abdominal pain, diarrhea or constipation, loss of appetite, heartburn, and/or dysphagia.

As the control group, 13 patients with the same inclusion and exclusion criteria and matched by sex, age, and BMI were selected from our database of approximately 1,000 patients with long-COVID. Controls were not treated with antihistamines, as they refused further pharmacological treatment.

Fatigue, brain fog, cardiovascular symptoms, and gastrointestinal manifestations were self-reported by the patients. Increased HR was assessed by a physician. The patients were treated with the following medications: fexofenadine 180 mg once daily taken before dinner and famotidine 40 mg once daily at bedtime. After 20 days of treatment, the patients were asked to answer four questions regarding the course of the symptoms mentioned at the time of enrolment. Each patient was asked to answer the following simple question for each symptom: is your symptom (1) unchanged or (2) improved or (3) disappeared or (4) worsened? As for increased heart rate, this was evaluated by the physician, but the patient was asked to answer questions about associated cardiovascular symptoms (palpitations, exercise intolerance, and chest pain). Untreated patients were asked to answer the same questions at baseline and 20 days later.

This study used information previously collected during normal care (with no intention to use it for research purposes at the time of collection). Written informed consent to be included in the study was obtained from each patient.

### Statistics

To find differences in continuous variables between the two study groups, the Student's *t*-test was used. Non-normally distributed variables were log-transformed before the analysis. Fisher's exact test or the chi-square test was performed for frequency comparisons. A *p*-value of less than 0.05 was considered statistically significant.

## Results

Table 1 depicts the demographic and clinical characteristics of treated and untreated patients at baseline. As shown, there were no differences in any of the investigated variables between the two study groups. Among the treated patients, 1 patient (a 44-year-old woman) showed two symptoms (fatigue and abdominal disorders), 2 patients (a 47-year-old woman and a 44-year-old woman) had three symptoms (fatigue, brain fog, and increased HR), and 11 patients had all four symptoms. Among the untreated patients (controls), 1 patient (a 58-year-old man) showed two symptoms (fatigue and abdominal disorders), whereas 4 patients had three symptoms (a 38-year-old woman with fatigue, increased HR, and abdominal disorders; a 38-year-old man, a 54-year-old man, and a 61-year-old woman had fatigue, brain fog, and abdominal disorders), and 8 patients had all four symptoms.

**Table 1:** Demographic and clinical characteristics of treated and untreated patients with long-COVID at baseline.

Variables	Treated patients (*n* = 14)	Untreated patients (*n* = 13)	*p*-Values
Age (years)	49.5 ± 11.5	47.3 ± 8.0	0.572
Females/Males (*n*)	9/5	8/5	1.000
BMI	25.6 ± 4.4	25.1 ± 2.7	0.359
Patients with two symptoms (*n*)	1	1	0.576
Patients with three symptoms (*n*)	2	4	
Patients with four symptoms (*n*)	11	8	

BMI, body mass index.

Table 2 shows the clinical outcomes of treated patients and controls. As shown, each symptom improved (in terms of improvement or disappearance of the symptom) in a significant proportion of the treated patients after 20 days of antihistamines treatment. Among them, fatigue fully vanished in six patients (43%), brain fog disappeared in six patients (43%), increased heart rate was no longer present in eight patients (57%), and abdominal disorders disappeared in eight patients (57%). Among controls, no symptom showed significant improvement and no symptoms disappeared in the untreated patients group.

**Table 2:** Clinical outcomes in 14 patients with long-COVID after 20 days of treatment with H1 and H2 receptor blockers, and in 13 untreated patients with long-COVID.

Symptoms	Treated patients (*n* = 14)	Untreated patients (*n* = 13)				
	At baseline (%)	Unchanged 20 days after (%)	*p*-Value	At baseline (%)	Unchanged 20 days after (%)	*p*-Value
Fatigue	100	21	<0.0001	100	85	0.4800
Brain fog	93	43	0.0127	85	70	0.6447
Increased HR	93	29	0.0013	92	85	1.0000
Abdominal disorders	79	14	0.0048	77	62	0.6727

HR, heart rate.

Table 3 shows the percentages of subjects with long-COVID with each symptom of long-COVID at baseline and after 20 days in the treated and untreated groups. At baseline, there were no differences in the prevalence of long-COVID and relative symptoms between the groups. The percentage of patients with fatigue, increased heart rate, and abdominal disorders was significantly lower in the treated than in the untreated group. Four in 14 treated patients (29%) did not show any symptoms of long-COVID after 20 days of treatment, while all untreated patients had long-COVID 20 days later. The magnitude of the difference was insufficient to reach statistical significance.

**Table 3:** Clinical outcomes in 14 patients with long-COVID after 20 days of treatment with H1/H2 receptor blockers and in 13 untreated patients with long-COVID.

	At baseline			20 days after		
	Treated patients (*n* = 14)	Untreated patients (*n* = 13)	*p*-Value	Treated patients (*n* = 14)	Untreated patients (*n* = 13)	*p*-Value
Long-COVID (%)	100	100	1.0000	71	100	0.0977
Unchanged fatigue (%)	100	100	1.0000	21	85	0.0018
Unchanged brain fog (%)	93	85	0.5955	43	70	0.2518
Increased HR (%)	93	92	1.0000	29	85	0.0063
Abdominal disorders (%)	79	77	1.0000	14	62	0.0183

HR, heart rate.

Neither patients nor controls showed worsening of any symptoms. No adverse effects due to treatment were observed.

## Discussion

Long-COVID has been broadly defined. According to the NICE (National Institute for Health and Care Excellence) definition, the term “long-COVID” should include both ongoing symptomatic COVID-19 (persistence of symptoms 4–12 weeks after the infection) and post-COVID syndrome (signs and symptoms developing during or after a SARS-Cov2 infection, persisting for more than 12 weeks and that are not explained by other diagnoses) (4). It is very difficult to know the exact incidence and prevalence of long-COVID in infected patients because of the different definitions, diagnostic criteria, targeted populations, and follow-up periods used in the studies. However, long-COVID is a common condition. Long-COVID may affect 10%–30% (or even more) of those who contracted COVID-19 (5). The prevalence of post-COVID may range from 3% to 11.7%, according to data from England (6). To our knowledge, there are no effective treatments for long-COVID; therefore, it can have a very long duration. A lot of therapies, including rehabilitation, nutritional counseling, psychological support, immunomodulator, acupuncture, antidepressants, steroids, beta-blockers, cough suppressants, non-steroidal anti-inflammatory drugs, analgesics, nutraceutical agents, vitamins, and anticoagulants, have been proposed and used to treat long-COVID, but their effectiveness is very relative (3, 4, 5, 8, 9).

Our preliminary data clearly show that blocking both histamine H1 and histamine H2 receptors may lead to the improvement or even the disappearance of some symptoms in a significant proportion of patients with long-COVID with symptoms attributed to MCA. Long-COVID even vanished in 29% of the treated patients.

These findings support the idea that MCA is involved in the pathophysiology of long-COVID, as suggested by some authors (11–13). In addition, our study may suggest that other pathophysiological mechanisms can play a role in the occurrence of long-COVID in non-responders to histamine blockers. Some patients did not have any significant benefit from the treatment with antihistamines, while others showed a partial response to the treatment. Immune dysregulation, autoimmunity, endothelial dysfunction, occult viral persistence, coagulation activation, and MCA have been proposed as potential mechanisms of long-COVID (6). However, long-COVID has several subtypes and, therefore, is not a homogeneous disease (5). This implies that each subtype may be caused by specific mechanisms (5, 7–9). Therefore, the classification of long-COVID in subtypes and the identification of their corresponding mechanisms by specific biomarkers may permit tailored treatments. Unfortunately, specific biomarkers and recognized classifications are lacking at present.

Mechanisms involved in the pathophysiology of long-COVID have also been described in patients with acute COVID-19 such as direct damage to infected cells via ACE2 receptors (14), coagulative abnormalities (14, 15), autoimmune mechanisms (16), oxidative stress (17), endothelial dysfunction (18), and MCA (10). In acute COVID-19, these mechanisms are usually linked to hyperinflammation that mediates clinical presentation, course, and prognosis (10). Hyperinflammation is an excessive inflammatory response of the host, called “cytokine storm”, which includes proinflammatory cytokines and chemokines released by immune effector cells, ultimately resulting in tissue damage and organ failure (10). Mast cells may play a role in cytokine storm (10). Specific studies should clarify whether the persistence of one or more mechanisms involved in acute COVID-19 can lead to specific subtypes of long-COVID when hyperinflammation disappears.

Our findings warrant further support by larger studies. However, it is worth noting that a “symptomatic” therapy with H1 and H2 receptor blockers is useful, as it can rapidly improve important disorders related to long-COVID, as already suggested (13). Specific studies should test not only antihistamines but also stabilizers of the mast cells as potential treatments for long-COVID if the MCA involvement is confirmed. Some trials with montelukast are ongoing and may provide interesting solutions (19). In addition, targeted studies should discover biomarkers for identifying patients with clinical presentations of long-COVID caused by MCA. That would allow clinicians to administer personalized therapeutical strategies. In patients with recognized MCA, in addition to pharmacological treatment, other measures should be adopted; patients may follow a specific “low histamine” diet (20) and their practice of physical activity should avoid an excessive production of histamine (21).

The effects that MCA can have on the cardiovascular system have been extensively described (23). In our cohort of patients with long-COVID, the benefits of the treatment with antihistamines appear to be particularly interesting regarding cardiovascular symptoms. The main cardiovascular manifestations are due to the pathological production and release of many mediators, such as histamine, tryptase, vasoactive intestinal polypeptide, and phospholipid metabolites, that usually cause tachycardia and hypotension (23). Beta-blockers are often used to reduce tachycardia, especially in patients with normal blood pressure. However, this therapy usually determines a further increase in heart rate, as experienced by many of our patients. Indeed, beta-blockers do not act on the mechanisms by which MCA increases heart rate, but they may even cause a blood pressure decrease with a consequent increase in heart rate via baroreflex activation (24). On the contrary, the blockage of H1 and H2 receptors permits a reduction in heart rate and an increase in blood pressure.

The present study has several limitations. The main limitation is the retrospective analysis. However, we used rigorous inclusion and exclusion criteria to explore the effectiveness of specific therapy in a well-characterized population. Controls were matched with treated patients. Then, we enrolled only patients with persistent long-COVID who did not respond to other treatments. In addition, acute COVID-19 and SARS-CoV2 infections were well-documented. Furthermore, none of our patients complained of any symptom attributed to long-COVID before the SARS-CoV2 infection, and none of the patients were vaccinated. We recruited only unvaccinated patients, considering that there is growing evidence that the SARS-CoV2 vaccine may affect the course of long-COVID (22). Another limitation of our study may be represented by our initial clinical choice of using antihistamines only in patients with symptoms of long-COVID attributable to MCA (12). This may explain the high rate of patients who presented with disappearance or improvement of the symptoms in a short time. Despite this, no controls showed significant improvement, even if the control group had the same features as the treated group. Except for heart rate measurement, all the patients' symptoms were self-reported. This may represent another study limitation. However, no biomarker or recognized sign is currently available for most clinical manifestations of long-COVID. No validated questionnaires were administered in this study, nevertheless, all the treated patients reported an improvement in their symptoms, and the therapeutical effects on HR were always documented by a physician. Moreover, none of the patients reported worsening of any symptoms of long-COVID, and no benefits were observed in untreated patients. Altogether, these results suggest the effectiveness of the treatment.

Our data confirm that blocking both H1 and H2 receptors can lead to a quick improvement in patients with long-COVID with specific symptoms attributed to MCA. These findings support the underlying role of MCA in the pathophysiology of long-COVID. Further larger-cohort studies are warranted to confirm these results.

## Data Availability

The raw data supporting the conclusions of this article will be made available by the authors, without undue reservation.

## References

[1] Gazzaruso C Carlo Stella N Mariani G Tamburlini A Garini P Freddi E Impact of anti-rheumatic drugs and steroids on clinical course and prognosis of COVID-19. Clin Rheumatol. (2020) 39:2475–7. 10.1007/s10067-020-05239-5 32548723 PMC7297548

[2] Pujia R Ferro Y Maurotti S Khoory J Gazzaruso C Pujia A The effects of COVID-19 on the eating habits of children and adolescents in Italy: a pilot survey study. Nutrients. (2021) 13:2641. 10.3390/nu13082641 34444801 PMC8400531

[3] Crook H Raza S Nowell J Young M Edison P. Long COVID-mechanisms, risk factors, and management. Br Med J. (2021) 374:n1648. 10.1136/bmj.n1648 34312178

[4] Mayer N Meza-Torres B Okusi C Delanerolle G Chapman M Wang W Developing a long COVID phenotype for post-acute COVID-19 in a national primary care sentinel cohort: an observational retrospective database analysis. JMIR Public Health Surveill. (2022). 8:e36989. 10.2196/36989 35861678 PMC9374163

[5] Yong SJ Liu S. Proposed subtypes of post-COVID-19 syndrome (or long-COVID) and their respective potential therapies. Rev Med Virol. (2021) 32:e2315. 10.1002/rmv.2315 34888989

[6] Castanares-Zapatero D Chalon P Kohn L Dauvrin M Detollenaere J Maertens de Noordhout C Pathophysiology and mechanism of long COVID: a comprehensive review. Ann Med. (2022) 54(1):1473–87. 10.1080/07853890.2022.2076901 35594336 PMC9132392

[7] Deer RR Rock MA Vasilevsky N Carmody L Rando H Anzalone AJ Characterizing long COVID: deep phenotype of a complex condition. EBioMedicine. (2021) 74:103722. 10.1016/j.ebiom.2021.103722 34839263 PMC8613500

[8] Greenhalgh T Knight M A'Court C Buxton M Husain L. Management of post-acute COVID-19 in primary care. Br Med J. (2020) 370:m3026. 10.1136/bmj.m3026 32784198

[9] Yong SJ. Long COVID or post-COVID-19 syndrome: putative pathophysiology, risk factors, and treatments. Infect Dis (Lond). (2021) 53(10):737–54. 10.1080/23744235.2021.1924397 34024217 PMC8146298

[10] Hafezi B Chan L Knapp JP Karimi N Alizadeh K Mehrani Y Cytokine storm syndrome in SARS-CoV-2 infections:a functional role of mast cells. Cells. (2021) 10:1761. 10.3390/cells10071761 34359931 PMC8308097

[11] Afrin LB Weinstock LB Molderings GJ. COVID-19 hyperinflammation and post-COVID-19 illness may be rooted in mast cell activation syndrome. Int J Infect Dis. (2020) 100:327–32. 10.1016/j.ijid.2020.09.016 32920235 PMC7529115

[12] Weinstock LB Brook JB Walters AS Goris A Afrin LB Molderings GJ. Mast cell activation symptoms are prevalent in long-COVID. Int J Infect Dis. (2021) 112:217–26. 10.1016/j.ijid.2021.09.043 34563706 PMC8459548

[13] Glynne P Tahmasebi N Gant V Gupta R. Long COVID following mild SARS-CoV-2 infection: characteristic T cell alterations and response to antihistamines. J Investig Med. (2022) 70(1):61–7. 10.1136/jim-2021-002051 34611034 PMC8494538

[14] Alnima T Mulder MMG van Bussel BCT Ten Cate H. COVID-19 coagulopathy: from pathogenesis to treatment. Acta Haematol. (2022) 145(3):282–96. 10.1159/000522498 35499460 PMC9059042

[15] Gazzaruso C Paolozzi E Valenti C Brocchetta M Naldani D Grignani C Association between antithrombin and mortality in patients with COVID-19. A possible link with obesity. Nutr Metab Cardiovasc Dis. (2020) 30(11):1914–9. 10.1016/j.numecd.2020.07.040 32907762 PMC7386389

[16] Gazzaruso C Carlo Stella N Mariani G Nai C Coppola A Naldani D High prevalence of antinuclear antibodies and lupus anticoagulant in patients hospitalized for SARS-CoV2 pneumonia. Clin Rheumatol. (2020) 39(7):2095–7. 10.1007/s10067-020-05180-7 32462425 PMC7251560

[17] Vollbracht C Kraft K. Oxidative stress and hyper-inflammation as Major drivers of severe COVID-19 and long COVID: implications for the benefit of high-dose intravenous vitamin C. Front Pharmacol. (2022) 13:899198. 10.3389/fphar.2022.899198 35571085 PMC9100929

[18] Ambrosino P Calcaterra IL Mosella M Formisano R D'Anna SE Bachetti T Endothelial dysfunction in COVID-19: a unifying mechanism and a potential therapeutic target. Biomedicines. (2022) 10(4):812. 10.3390/biomedicines10040812 35453563 PMC9029464

[19] Ceban F Leber A Jawad MY Yu M Lui LMW Subramaniapillai M Registered clinical trials investigating treatment of long COVID: a scoping review and recommendations for research. Infect Dis (Lond). (2022) 54(7):467–77. 10.1080/23744235.2022.2043560 35282780 PMC8935463

[20] San Mauro Martin I Brachero S Garicano Vilar E. Histamine intolerance and dietary management: a complete review. Allergol Immunopathol (Madr). (2016) 44(5):475–83. 10.1016/j.aller.2016.04.015 27590961

[21] Luttrell MJ Halliwill JR. The intriguing role of histamine in exercise responses. Exerc Sport Sci Rev. (2017) 45(1):16–23. 10.1249/JES.0000000000000093 27741023 PMC5161583

[22] Sivan M Greenhalgh T Milne R Delaney B. Are vaccines a potential treatment for long COVID? Br Med J. (2022) 377:o988. 10.1136/bmj.o988 35584848

[23] Kolck UW Haenisch B Molderings GJ. Cardiovascular symptoms in patients with systemic mast cell activation disease. Transl Res. (2016) 174:23–32.e1. 10.1016/j.trsl.2015.12.012 26775802

[24] Bernardi L Salvucci F Leuzzi S Radaelli A Calciati A Valle F Cardiovascular reflex changes preceding episodes of vasovagal syncope in paediatric subjects. Clin Sci (Lond). (1996) 91(Suppl):25–7. 10.1042/cs0910025supp 8813819

